# *Vital Signs*: Asthma in Children — United States, 2001–2016

**DOI:** 10.15585/mmwr.mm6705e1

**Published:** 2018-02-09

**Authors:** Hatice S. Zahran, Cathy M. Bailey, Scott A. Damon, Paul L. Garbe, Patrick N. Breysse

**Affiliations:** ^1^Division of Environmental Health Science and Practice (proposed), National Center for Environmental Health, CDC; ^2^Director, National Center for Environmental Health, Agency for Toxic Substances and Disease Registry, CDC.

**Background:** Asthma is the most common chronic lung disease of childhood, affecting approximately 6 million children in the United States. Although asthma cannot be cured, most of the time, asthma symptoms can be controlled by avoiding or reducing exposure to asthma triggers (allergens and irritants) and by following recommendations for asthma education and appropriate medical care.

**Methods:** CDC analyzed asthma data from the 2001–2016 National Health Interview Survey for children aged 0–17 years to examine trends and demographic differences in health outcomes and health care use.

**Results:** Asthma was more prevalent among boys (9.2%) than among girls (7.4%), children aged ≥5 years (approximately 10%) than children aged <5 years (3.8%), non-Hispanic black (black) children (15.7%) and children of Puerto Rican descent (12.9%) than among non-Hispanic white (white) children (7.1%), and children living in low income families (10.5%) than among those living in families with income ≥250% of the Federal Poverty Level (FPL) (approximately 7%). Asthma prevalence among children increased from 8.7% in 2001 to 9.4% in 2010, and then decreased to 8.3% in 2016. Although not all changes were statistically significant, a similar pattern was observed among subdemographic groups studied, with the exception of Mexican/Mexican-American children, among whom asthma prevalence increased from 5.1% in 2001 to 6.5% in 2016.

Among children with asthma, the percentage who had an asthma attack in the past 12 months declined significantly from 2001 to 2016. Whereas asthma prevalence was lower among children aged 0–4 years than among older children, the prevalence of asthma attacks (62.4%), emergency department or urgent care center (ED/UC) visits (31.1%), and hospitalization (10.4%) were higher among children with asthma aged 0–4 years than among those aged 12–17 years (44.8%, 9.6%, and 2.8%, respectively).

During 2013, children with asthma aged 5–17 years missed 13.8 million days of school per year (2.6 days per child). Compared with 2003, in 2013, the prevalence of adverse health outcomes and health care use were significantly lower and the prevalence of having an action plan to manage asthma was higher.

**Conclusions and Implications for Public Health Practice:** Asthma remains an important public health and medical problem. The health of children with asthma can be improved by promoting asthma control strategies, including asthma trigger reduction, appropriate guidelines-based medical management, and asthma education for children, parents, and others involved in asthma care.

## Introduction

Asthma is a common chronic lung disease of children that causes repeated episodes of wheezing, breathlessness, chest tightness, and nighttime or early morning coughing (*1*). These symptoms can often be controlled by avoiding or reducing asthma triggers (allergens and irritants) and by following recommendations for appropriate medical care (initiating asthma control medications or adjusting the current treatment regimen when needed) (*1*,*2*).

A 2012 CDC National Surveillance of Asthma report showed an increasing trend in asthma prevalence among children between 2001 and 2010, with children experiencing more asthma attacks and emergency visits than did adults (*3*). Asthma is more common among some children than others. Boys, children aged ≥5 years, black children and children of Puerto Rican descent, and children living in households with income of <100% of FPL had higher prevalence than did girls, children aged <5 years, white children, and children living in households with income >250% FPL (*3*,*4*). Asthma-related hospitalizations were 3.6 times higher and emergency department visits were 3 times higher among black children than among white children (*4*).

Uncontrolled asthma results in significant costs to families and society when asthma exacerbations result in medical encounters, lost school days, and reduced productivity. The cost of asthma for children varies by state. In 2012, the median annual medical cost of asthma was $983 per child (ranging from $833 in Arizona to $1,121 in Michigan) for all payers (*5*).

Because of changing physical, social, and economic environments and medical management of asthma at individual and population levels over time (*6*,*7*), there is a need to update prevalence estimates and to reassess demographic differences in health outcomes and health care use to better define the current burden of asthma overall and among subpopulations. This report reviews the current state of asthma among U.S. children aged 0–17 years and related health outcomes, health care use, and asthma care and management.

## Methods

To describe asthma status and to assess trends and demographic differences in self-reported health outcomes, health care use, and asthma care and management among children aged 0**–**17 years, CDC analyzed annual core* data (2001–2016) and periodic asthma supplemental^†^ data (2003, 2008, and 2013) from the National Health Interview Survey (NHIS).

The NHIS, conducted by CDC’s National Center for Health Statistics (NCHS), is a cross-sectional household interview survey of the U.S. civilian noninstitutionalized population in 50 states and the District of Columbia. NHIS uses a multistage, clustered sample design, and applies sampling weights to account for household nonresponse and oversampling of blacks, Hispanics, and Asians to produce national estimates for a variety of health indicators (the sampling design was changed in 2016, and oversampling of these groups was not conducted during that year). NHIS collects additional data on asthma (e.g., routine care visits, hospitalization, missed school days, self-management education, and asthma medication use [rescue and control medications]) every 5 years (i.e., 2003, 2008, and 2013; https://www.cdc.gov/nchs/nhis/about_nhis.htm).

In 2016, persons aged 0–17 years accounted for 11,107 of NHIS respondents, including 960 (8.3%) who had current asthma. Children were considered to have current asthma if proxy adults answered “yes” to the following two questions: “Has a doctor or other health professional ever told you that [child] had asthma?” and “Does [child] still have asthma?” (*3*,*4*). Trends in prevalence of current asthma (asthma) and asthma attack were assessed. Among children with asthma, demographic (age, sex, race/ethnicity, income status, and U.S. Bureau of the Census geographic region) differences in self-reported school absenteeism, asthma attack, and health care use because of asthma (routine care visit, ED/UC visit, and hospitalization) in the past 12 months were assessed. Prevalences of asthma attack and ED/UC visit were defined as the percentage of children with current asthma who experienced an asthma attack and had an ED/UC visit because of an asthma attack in the past 12 months, respectively. School absenteeism was defined as one or more missed school days by a child aged 5–17 years in the past 12 months. NHIS 2003, 2008, and 2013 data were also analyzed to assess changes in health care use (asthma-related routine care visit and hospitalization in the past 12 months) and asthma care status (ever received any of the 6-component asthma self-management education,^§^ and asthma medication use [rescue medication and asthma control medication] in the past 3 months). Additional information is available at https://www.cdc.gov/nchs/nhis/index.htm.

Statistical software was used for analysis to account for the complex sampling design. Trends in prevalence of current asthma and asthma attack during 2001–2016 were assessed using Joinpoint software from the National Cancer Institute (NCI) (*8*), which characterizes trends as joined linear segments. All stated comparisons between demographic groups were evaluated by using two-sided significance tests with statistical significance defined as p<0.05. Relative standard error (RSE), defined as standard error divided by prevalence estimate, was used as a measure of an estimate’s reliability (an RSE <0.30 indicates a reliable estimate) (*3*).

## Results

During 2016, asthma affected boys (9.2%) more than girls (7.4%), children aged 5–11 years (9.6%) and 12–17 years (10.5%) more than children aged 0–4 years (3.8%), black children (15.7%) and children of Puerto Rican descent (12.9%) more than white children (7.1%), and children living in families with income of less than 100% FPL (10.5%) more than those living in families with income of ≥250% FPL (250% to <450% FPL: 6.9%; ≥450% FPL: 6.7%). However, current asthma prevalence did not differ significantly by U.S. Census region (Table 1) or metropolitan statistical area (MSA).

**TABLE 1:** Demographic characteristics and prevalence of current asthma among U.S. children aged 0–17 years — National Health Interview Survey, 2016

Demographic characteristic	Sample size* (U.S. children aged 0–17) (%)^†^	Prevalence of current asthma (6.1 million)		p-value^§^
		Sample size* (children with current asthma)	% (95% CI)^†^	
**Total**	11,107 (100)	960	8.3 (7.7–9.0)	—^¶^
**Sex**				
Boys	5,743 (51.0)	564	9.2 (8.3–10.3)	<0.01
Girls	5,364 (49.0)	396	7.4 (6.6–8.3)	Referent
**Age group (yrs)**				
0–4	3,042 (27.2)	111	3.8 (3.0–4.9)	Referent
5–11	4,076 (39.0)	421	9.6 (8.5–10.8)	<0.0000
12–17	3,989 (33.8)	428	10.5 (9.4–11.8)	<0.0000
**Race/Ethnicity**				
White, non-Hispanic	6,110 (51.5)	445	7.1 (6.3–8.0)	Referent
Black, non-Hispanic	1,286 (13.5)	201	15.7 (13.6–18.2)	<0.0000
Other, non-Hispanic	1,305 (10.1)	126	8.8 (6.9–11.1)	—^¶^
Hispanic	2,406 (24.9)	188	6.7 (5.5–8.2)	—^¶^
Puerto Rican	243 (2.5)	40	12.9 (8.9–18.4)	<0.05
Mexican/Mexican American	1,518 (15.9)	111	6.5 (5.0–8.5)	—^¶^
All other Hispanics	645 (6.5)	37	4.9 (3.2–7.5)	—^¶^
**Ratio of family income to poverty threshold**				
<100% FPL	1,813 (19.3)	202	10.5 (8.8–12.4)	<0.001
100% to <250% FPL	3,431 (32.2)	350	9.4 (8.2–10.7)	<0.01
250% to <450% FPL	2,943 (25.0)	210	6.9 (5.8–8.1)	—^¶^
≥450% FPL	2,919 (23.5)	197	6.7 (5.7–8.0)	Referent
**U.S. Census region**				
Northeast	1,808 (18.0)	167	8.2 (6.7–10.2)	—^¶^
Midwest	2,294 (21.4)	175	7.8 (6.6–9.2)	—^¶^
South	3,938 (36.8)	369	9.2 (8.1–10.4)	—^¶^
West	3,067 (23.7)	249	7.7 (6.5–9.0)	—^¶^

**Abbreviations:** CI = confidence interval; FPL = Federal Poverty Level (based on family income and family size, using the U.S. Census Bureau’s poverty thresholds).

* Unweighted sample size.

^†^ Weighted percentage and confidence interval.

^§^ p-value testing for differences in current asthma prevalence between intended group and corresponding referent group.

^¶^ Not statistically significant.

Asthma prevalence among children aged 0–17 years increased from 8.7% in 2001 to 9.4% in 2010, and then decreased to 8.3% in 2016. Although not all changes were statistically significant, a similar pattern was observed among all sex, age, and racial/ethnic groups studied, except for Mexican/Mexican-American children, among whom asthma prevalence increased from 5.1% in 2001 to 6.5% in 2016.

In 2013 and 2016, nearly 54% of children with asthma were reported to have had ≥1 asthma attack, 71.1% had routine care visits, 4.7% were hospitalized, 16.7% had an ED/UC visit because of an asthma attack, and 49.0% of school-age children with asthma missed one or more school days (Table 2). Having an asthma attack, missing school days, and having health care visits because of asthma (routine care visits and hospitalizations) did not differ by sex, race/ethnicity, and U.S. census region. However, the prevalence of asthma attacks, hospitalizations, and ED/UC visits were higher among children aged 0–4 years than among those aged 12–17 years and ED/UC visits were higher among black children (22.5%) than among white children (12.2%) (Table 2).

**TABLE 2:** Health outcomes and healthcare use by demographic characteristics among children with current asthma — National Health Interview Survey, 2013 and 2016

Demographic characteristic	Missed school days* (ages 5–17) (2013 NHIS)	Asthma attacks* (2016 NHIS)	Health care use		
			Routine care visits* (2013 NHIS)	Hospitalized* (2013 NHIS)	ED/UC visits*^,†^ (2016 NHIS)
	% (95% CI)^§^	% (95% CI)^§^	% (95% CI)^§^	% (95% CI)^§^	% (95% CI)^§^
**Total**	49.0 (44.9–53.0)	53.7 (49.8–57.7)	71.1 (68.0–74.1)	4.7 (3.4–6.5)	16.7 (13.6–20.2)
**Sex**					
Boys	51.3 (46.2–56.4)	54.6 (49.1–60.0)	72.0 (67.7–75.9)	5.3 (3.6–7.6)	17.8 (13.8–22.6)
Girls	46.0 (39.7–52.5)	52.7 (46.3–58.9)	70.0 (64.3–75.1)	4.0 (2.2–7.2)	15.2 (11.0–20.6)
**Age group (yrs)**					
0–4	NA	62.4 (50.8–72.7)^¶^	78.2 (68.0–85.8)	10.4 (6.1–17.4)**	31.1 (20.9–43.6)^††^
5–11	52.0 (46.1–57.8)	59.8 (53.8–65.6)^§§^	73.0 (67.1–78.2)	4.8 (3.1–7.3)	19.4 (14.7–25.1)^¶¶^
12–17	45.5 (40.1–51.0)	44.8 (39.0–50.9)	66.6 (61.4–71.4)	2.8 (1.2–6.3)	9.6 (6.5–13.9)
**Race/Ethnicity**					
White, non-Hispanic	43.8 (37.6–50.3)	53.9 (47.7–60.0)	72.2 (67.2–76.7)	3.3 (1.7–6.2)	12.2 (8.6–17.1)
Black, non-Hispanic	52.7 (44.6–60.7)	53.1 (44.7–61.4)	72.2 (65.1–78.3)	6.8 (3.9–11.7)	22.5 (15.8–31.0)***
Other, non-Hispanic	50.3 (37.0–63.6)	63.6 (52.4–73.5)	59.2 (48.0–69.6)	4.6 (1.5–13.2)	22.1 (13.7–33.6)
Hispanic	56.5 (49.0–63.7)	48.9 (40.5–57.4)	72.5 (65.4–78.7)	5.8 (3.5–9.5)	16.2 (9.8–25.5)
Puerto Rican	50.6 (30.7–70.3)	46.5 (28.8–65.2)	72.2 (48.4–87.7)	6.5 (2.4–16.6)	8.8 (3.0–23.0)^†††^
Mexican/Mexican-American	57.3 (47.6–66.4)	46.1 (35.8–56.7)	75.6 (68.0–81.8)	7.0 (3.6–13.2)	16.0 (8.4–28.4)^†††^
All other Hispanics	59.2 (46.3–71.0)	60.5 (39.5–78.3)	66.5 (50.6–79.3)	2.8 (0.9–8.8)	24.1 (8.9–50.9)^†††^
**Ratio of family income to poverty threshold** ^§§§^					
<100% FPL^¶^	54.8 (47.3–62.1)	53.8 (45.3–62.1)	74.4 (68.0–79.8)	7.2 (4.7–10.8)	21.1 (13.9–30.6)
100% to <250% FPL	47.7 (40.4–55.0)	51.9 (45.0–58.7)	73.8 (67.1–79.5)	4.4 (2.2–8.9)	18.7 (13.9–24.7)
250% to <450% FPL	45.1 (36.8–53.7)	53.9 (44.8–62.6)	61.9 (53.2–69.8)	2.1 (0.4–10.4)	11.3 (6.7–18.5)
≥450% FPL	46.4 (38.0–55.0)	57.1 (47.5–66.2)	71.9 (63.6–78.9)	4.0 (1.3–11.4)	13.1 (7.6–21.6)
**U.S. Census region**					
Northeast	55.4 (45.8–64.6)	55.9 (47.6–64.0)	77.5 (71.1–82.8)	3.4 (1.5–7.5)	12.8 (7.7–20.5)
Midwest	38.4 (31.3–46.0)	52.5 (42.9–61.9)	67.3 (58.4–75.0)	4.2 (2.1–8.4)	18.7 (12.0–28.0)
South	49.1 (42.1–56.1)	51.2 (45.2–57.1)	71.1 (66.3–75.5)	6.0 (3.7–9.7)	17.0 (12.5–22.6)
West	53.4 (45.5–61.1)	57.8 (48.7–66.5)	69.6 (63.0–75.4)	3.9 (2.0–7.3)	17.4 (10.9–26.6)

**Abbreviations:** CI = confidence interval; ED/UC = emergency department/urgent care; FPL = Federal Poverty Level; NA = not available; NHIS = National Health Interview Survey.

* Self-reported asthma related missed school days, asthma attacks, routine care visits, and if hospitalized and had an ED/UC visit in the past 12 months.

^†^ ED/UC visits were among children with current asthma who experienced an asthma attack.

^§^ Weighted percentage.

^¶^ p-value <0.01 testing for differences in asthma attack prevalence between children aged 0–4 and aged 12–17.

** p-value <0.05 testing for differences in hospitalization between children aged 0–4 and aged 12–17.

^††^ p-value <0.001 testing for differences in ED/UC visit prevalence between children aged 0–4 and aged 12–17.

^§§^ p-value <0.001 testing for differences in asthma attack prevalence between children aged 5–11and aged 12–17.

^¶¶^ p-value <0.01 testing for differences in ED/UC visit prevalence between children aged 5–11 and aged 12–17.

*** p-value <0.05 testing for differences in ED/UC visit prevalence between non-Hispanic black children and non-Hispanic white children.

^†††^ Relative Standard Errors are >30% indicating “unreliable” estimates.

^§§§^ FPL is federal poverty level. Based on family income and family size, using the U.S. Census Bureau’s poverty thresholds.

During 2001–2016, the percentage of children with asthma who experienced an asthma attack decreased significantly, from 61.7% in 2001 to 53.7% in 2016 (Figure). A significant decline in asthma attacks was experienced across all sex, age, and racial/ethnic groups.

**FIGURE:**
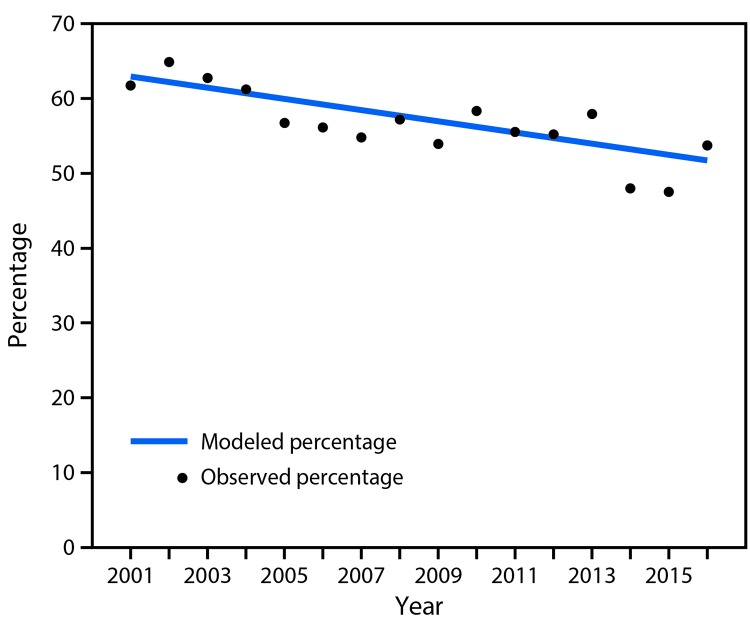
Percentage of asthma attacks among children aged 0–17 years with current asthma, by year — National Health Interview Survey, 2001–2016

Assessment of asthma self-management education found that 50.8% of children with asthma received an asthma action plan, 11.0% were taking a class to learn how to manage their asthma, 76.0% were taught how to recognize early signs of an asthma attack, 80.0% were taught how to respond to an asthma attack, 50.6% were taught how to use a peak flow meter (a portable, handheld device that is used to measure how well air moves out of the lungs), and 46.4% received advice on environmental control in 2013. Compared with 2003, the percentages of children with asthma who were hospitalized because of asthma and, among school-aged children with asthma, the percentage with missed school days were significantly lower in 2013, while the percentage having an action plan to manage asthma was higher (Table 3). Similar to estimates in 2003, in 2013, 94.4% of children with asthma had health insurance coverage, and 6.4% could not afford prescription medicine during the past 12 months. In 2013, nearly 68% of children with asthma were taking asthma rescue medications and 55.2% had taken asthma control medicine in the past 3 months. In addition, approximately 9% of children with asthma overused rescue medications (i.e., used more than three disks or canisters of quick relief inhaler medication in the past 3 months) and 30.1% were taking asthma control medications every day or almost every day as recommended, with 25.1% reporting taking them less often (Table 3). Having received self-management education and use of asthma control prescription medication did not differ by race/ethnicity. However, among children with asthma who were taking asthma control medicine during the preceding 3 months, the percentage of children using asthma control prescription medicine regularly as prescribed declined significantly from 65.7% in 2003 to 54.5% in 2013 (p<0.01) (Table 3).

**TABLE 3:** Prevalence of selected characteristics among children aged 0–17 years with current asthma — National Health Interview Survey, 2003, 2008, and 2013

Characteristic	2003	2008	2013	p-value (significant difference in estimates [2003 versus 2013])
	% (95% CI)*	% (95% CI)*	% (95% CI)*	
Mean no. of missed school days^†^ (95% CI)	4.2 (3.6–4.9)	3.3 (2.5–4.1)	2.6 (2.1–3.2)	p<0.001
Missed school days^†^	61.4 (56.2–66.4)	59.6 (52.5–66.3)^§^	49.0 (44.9–53.0)	p<0.001
Hospitalized^†^ because of asthma	9.6 (7.3–12.5)	8.0 (5.3–12.1)	4.7 (3.4–6.5)	p<0.01
Have health insurance coverage	93.1 (90.8–94.8)	93.9 (91.3–95.7)	94.4 (92.5–95.9)	—^¶^
Cannot afford prescription medicine	6.1 (4.6–8.2)	5.9 (4.2–8.2)	6.4 (4.6–8.7)	—^¶^
Self-management education**				
Given an action plan	39.5 (36.1–43.0)	44.3 (39.8–48.9)^††^	50.8 (46.8–54.7)	p<0.0001
Taken a class to learn how to manage their asthma	16.1 (13.8–18.8)	12.5 (9.8–15.9)	11.0 (8.9–13.5)	p<0.01
Taught to recognize early signs and symptoms of an asthma attack	72.4 (69.0–75.6)	72.1 (67.9–76.0)	76.0 (72.4–79.2)	—^¶^
Taught to respond to an asthma attack	77.5 (74.3–80.4)	78.3 (74.5–81.8)	80.0 (76.7–82.9)	—^¶^
Taught to use a peak flow meter	56.8 (52.8–60.7)	49.4 (44.8–54.0)^††^	50.6 (46.8–54.3)	p<0.05
Given advice on environmental control	53.1 (49.6–56.5)	50.6 (46.0–55.1)	46.4 (42.5–50.3)	p<0.05
**Rescue asthma medication use in past 3 months**				
Rescue asthma medication use	59.8 (56.1–63.3)	59.4 (54.9–63.8)^§§^	67.7 (64.2–71.0)	p<0.01
Overuse of rescue asthma medication in past 3 months^¶¶^	9.3 (7.4–11.6)	8.3 (6.2–10.9)	8.8 (6.4–11.9)	—^¶^
Asthma control medication use during past 3 months***				
Use asthma control medication	49.8 (46.2–53.4)	53.2 (48.6–57.7)	55.2 (51.4–58.9)	—^¶^
Use every day or almost every day	32.7 (29.4–36.0)	31.5 (27.5–35.8)	30.1 (26.4–34.0)	—^¶^
Use less often	17.1 (14.6–19.8)	21.7 (18.0–25.8)	25.1 (22.1–28.5)	p<0.001
Never used	50.3 (46.7–53.9)	46.8 (42.3–51.4)	44.8 (41.1–48.6)	—^¶^
**Use asthma control medication**				
Use every day or almost every day	65.7 (61.0–70.1)	59.2 (52.9–65.3)	54.5 (49.1–59.7)	p<0.01
Use less often	34.3 (29.9–39.0)	40.8 (34.7–47.1)	45.5 (40.3–50.9)	p<0.01

**Abbreviation:** CI = confidence interval.

* Weighted percentage.

^†^ Self-reported asthma related missed school days and hospitalization in the past 12 months.

^§^ p-value <0.05 testing for differences in estimates for “Missed school days” between 2008 and 2013.

^¶^ Not statistically significant.

** Self-management education related questions were asked every 5 years and if participants were ever been provided these type of education.

^††^ p-value <0.05 testing for differences in estimates for “Given an action plan” between 2008 and 2013, and for “Taught how to use a peak flow meter” between 2003 and 2008.

^§§^ p-value <0.01 testing for differences in estimates between 2008 and 2013.

^¶¶^ Use of more than three canisters or disks of quick relief inhaler (asthma rescue medication) by a child taking asthma rescue medications in the past 3 months.

*** If child taking an asthma control medication and how often (i.e., every day or almost every day, less often, or never) in the past 3 months.

## Conclusions and Comments

Although asthma still affects some children more than others, the findings in this report are somewhat encouraging. The prevalence of asthma and asthma attacks have decreased in recent years (since 2010 and 2001, respectively), fewer children with asthma reported missed school days and hospitalizations because of asthma, and more children with asthma received a written asthma action plan during 2013 than did during 2003. Among children with asthma, asthma attacks, hospitalizations, and ED/UC visits were more prevalent among children aged 0–4 years than among children aged 12–17 years. This might be partially explained by more frequent viral respiratory infections among this age group. These infections are the most common precipitants of asthma symptoms and hospitalizations among this age group (*9*).

The findings in this report indicate that more children with asthma received an asthma action plan, were taught how to recognize early signs of an asthma attack, and were taught how to respond to an asthma attack in 2013 than in 2003. However, in 2013 only half (51%) of children with asthma received an asthma action plan and less than half (46%) received advice on environmental control, indicating a need for further improvement in these areas, given that multicomponent self-management education programs, including an written asthma action plan (*1*,*10*,*11*); educating healthcare providers (*12*) can improve asthma-related health outcomes and reduce unnecessary health care use.

Access to and adherence to guidelines-based medical care, including prescribing inhaled corticosteroids, is a key component of effective asthma care (*1*,*13*,*14*). The findings show that just over half (54.5%) of children with asthma who were taking asthma control medications were taking them regularly as prescribed, indicating a need for further improvement in medication adherence.

The findings in this report are subject to at least two limitations. First, because NHIS is a cross-sectional survey, it provides prevalence estimates and associations, but cannot determine causal associations. Second, NHIS data are based on adult proxy responses for children; therefore, the findings might be biased because of inaccurate recall or the social desirability of providing positive responses.

Asthma remains an important public health and medical problem. Some progress has been made in providing asthma education and in decreasing adverse health outcomes. The health of children with asthma can be further improved by promoting asthma control strategies, including asthma trigger reduction, appropriate guidelines-based medical management, and asthma education for children, parents, and others involved in asthma care. The CDC National Asthma Control Program (https://www.cdc.gov/asthma/nacp.htm) works with 25 funded state and territorial grantees and four nongovernmental organizations to engage persons with asthma, their families, schools, communities, and health care providers to achieve better care and better health outcomes and to decrease unnecessary asthma-related emergency department and urgent care visits and hospitalizations.

**Key Points**

• One in 12 children aged 0–17 years had asthma in 2016.

• Asthma was more prevalent among boys, non-Hispanic black children, children of Puerto Rican descent, and children from low-income households.

• The percentage of children with asthma who had an asthma attack during the preceding year declined from 2001 to 2016. Even so, approximately half of children with diagnosed asthma had one or more asthma attack in 2016.

• Children with asthma had fewer missed school days and hospitalizations in 2013 compared with 2003.

• Approximately 55% children with asthma were taking asthma control prescription medicines during the preceding 3 months. Among children who were taking asthma control medicines, only 54.5% of them were taking control medicines regularly as prescribed, which was significantly lower than during 2003.

• The health of children with asthma can be further improved by promoting asthma control strategies, including asthma trigger reduction, appropriate guidelines-based medical management, and asthma education for children, parents, and others involved in asthma care.

• Additional information is available at https://www.cdc.gov/vitalsigns/.

## Footnotes

* Core data include sociodemographic characteristics and information on health conditions, health care access and utilization, health behaviors and risk factors.

† Supplemental modules collect data on new topics or more detailed information on core topics; can change from year to year; and are designed to meet department goals and objectives.

§ The 6-component asthma self-management education includes 1) having been given an action plan, 2) having taken a class to learn how to manage asthma, 3) having been taught to recognize early signs and symptoms of an asthma attack, 4) having been taught how to respond to an asthma attack, 5) having been taught to use a peak flow meter, and 6) having received advice on environmental control.
